# Perspective having a Centre of Expertise that covers more than one rare disease

**DOI:** 10.1186/1750-1172-9-S1-O2

**Published:** 2014-11-11

**Authors:** John R Ostergaard

**Affiliations:** 1 Centre for Rare Diseases, Aarhus University Hospital, Aarhus, DK 8200 Aarhus N, Denmark

## 

The vast majority of rare diseases affect more than one organ of the body, and eighty-five percent of the patients have symptoms within the initial five years of life. In childhood, the medical specialty, i.e. pediatrics, is not confined to one organ, but by age. In adulthood, the medical specialties are defined by an organ (e.g. heart, eye, urinary system, etc). As rare diseases do not respect borders for either age or organ, the organizational approach of healthcare for rare diseases need to take that into consideration.

At the Centre for Rare Diseases at Aarhus University Hospital, Denmark we want to use that “cradle to grave” approach. At the Centre, we see more than 1200 different patients, distributed across more than 100 different diagnoses. The core-staff consists of three pediatricians who all have clinical, diagnostic, treatment and coordinating tasks, and who either see the patients alone or by having joint clinical consultations with a variety of organ-related specialists.

A cooperative medical relationship for one specific diagnosis, for example, Tuberous Sclerosis, can also be used taking care of patients having Neurofibromatosis or von Hippel Lindau disease. At a certain time, they all may need care from a neurosurgeon, an ophthalmologist, a dermatologist, etc.. We make sure that there are one or few dedicated people involved from each specialty, and in this way we manage to get a firmly close-knitted established team in which the personal from the different medical specialties know each other, and are tuned to take care of patients with different diseases having similar medical challenges. We think, that this approach gives a lot of synergy effects and “spin of”, not only in the setting of treatment, but also as regard the diagnostic set up, and also as regard research.

Our setup provides us with significant challenges as well. It is difficult to be an expert regarding too many diagnoses, and as we continuously have to include new patients, and the patients cannot we processed to others, we have a great accumulation of patients. However, patients with rare diseases need treatment and care anyway, and we will try to meet this challenge with organizing ourselves scattered across the University Hospital in a Core Centre, primarily doing the diagnostic set-up, and with satellite centers, taking care of the treatment, placed at different organ-related highly specialized, but mutually coordinated, departments.

